# Chlorhexidine for facility-based umbilical cord care: EN-BIRTH multi-country validation study

**DOI:** 10.1186/s12884-020-03338-4

**Published:** 2021-03-26

**Authors:** Sojib Bin Zaman, Abu Bakkar Siddique, Harriet Ruysen, Ashish KC, Kimberly Peven, Shafiqul Ameen, Nishant Thakur, Qazi Sadeq-ur Rahman, Nahya Salim, Rejina Gurung, Tazeen Tahsina, Ahmed Ehsanur Rahman, Patricia S. Coffey, Barbara Rawlins, Louise T. Day, Joy E. Lawn, Shams El Arifeen, EN-BIRTH Study Group, Md. Ayub Ali, Bilkish Biswas, Rajib Haider, Md. Abu Hasanuzzaman, Md. Amir Hossain, Ishrat Jahan, Rowshan Hosne Jahan, Jasmin Khan, M. A. Mannan, Tapas Mazumder, Md. Hafizur Rahman, Md. Ziaul Haque Shaikh, Aysha Siddika, Taslima Akter Sumi, Md. Taqbir Us Samad Talha, Evelyne Assenga, Claudia Hanson, Edward Kija, Rodrick Kisenge, Karim Manji, Fatuma Manzi, Namala Mkopi, Mwifadhi Mrisho, Andrea Pembe, Jagat Jeevan Ghimire, Regina Gurung, Elisha Joshi, Avinash K. Sunny, Naresh P. KC, Nisha Rana, Shree Krishna Shrestha, Dela Singh, Parashu Ram Shrestha, Nishant Thakur, Hannah Blencowe, Sarah G. Moxon, Agbessi Amouzou, Tariq Azim, Debra Jackson, Theopista John Kabuteni, Matthews Mathai, Jean-Pierre Monet, Allisyn Moran, Pavani Ram, Barbara Rawlins, Jennifer Requejo, Johan Ivar Sæbø, Florina Serbanescu, Lara Vaz

**Affiliations:** 1 Maternal and Child Health Division, International Centre for Diarrhoeal Disease Research, Bangladesh (icddr,b), 68 Shahid Tajuddin Ahmed Sarani, Mohakhali, Dhaka, Bangladesh; 2 The Maternal, Adolescent, Reproductive, & Child, Health (MARCH) Centre, London School of Hygiene & Tropical Medicine, London, UK; 3 Department of Women’s and Children’s Health, Uppsala University, Uppsala, Sweden; 4 Florence Nightingale Faculty of Nursing, Midwifery & Palliative Care, King’s College London, London, UK; 5 Research Division, Golden Community, Lalitpur, Nepal; 6 Department of Paediatrics and Child Health, Muhimbili University of Health and Allied Sciences, Dar es Salaam, Tanzania; 7 Department of Health Systems, Impact Evaluation and Policy, Ifakara Health Institute, Dar es Salaam, Tanzania; 8 PATH, Seattle, WA USA; 9 Maternal and Child Survival Program, jhpiego, Baltimore, MD USA

**Keywords:** Birth, Newborn, Coverage, Validity, Survey, Hospital records, Health management systems, 7.1% chlorhexidine, Umbilical cord care, Neonatal sepsis

## Abstract

**Background:**

Umbilical cord hygiene prevents sepsis, a leading cause of neonatal mortality. The World Health Organization recommends 7.1% chlorhexidine digluconate (CHX) application to the umbilicus after home birth in high mortality contexts. In Bangladesh and Nepal, national policies recommend CHX use for all facility births. Population-based household surveys include optional questions on CHX use, but indicator validation studies are lacking. The *Every Newborn* Birth Indicators Research Tracking in Hospitals (EN-BIRTH) was an observational study assessing measurement validity for maternal and newborn indicators. This paper reports results regarding CHX.

**Methods:**

The EN-BIRTH study (July 2017–July 2018) included three public hospitals in Bangladesh and Nepal where CHX cord application is routine. Clinical-observers collected tablet-based, time-stamped data regarding cord care during admission to labour and delivery wards as the gold standard to assess accuracy of women’s report at exit survey, and of routine-register data. We calculated validity ratios and individual-level validation metrics; analysed coverage, quality and measurement gaps. We conducted qualitative interviews to assess barriers and enablers to routine register-recording.

**Results:**

Umbilical cord care was observed for 12,379 live births. Observer-assessed CHX coverage was very high at 89.3–99.4% in all 3 hospitals, although slightly lower after caesarean births in Azimpur (86.8%), Bangladesh. Exit survey-reported coverage (0.4–45.9%) underestimated the observed coverage with substantial “don’t know” responses (55.5–79.4%). Survey-reported validity ratios were all poor (0.01 to 0.38). Register-recorded coverage in the specific column in Bangladesh was underestimated by 0.2% in Kushtia but overestimated by 9.0% in Azimpur. Register-recorded validity ratios were good (0.9 to 1.1) in Bangladesh, and poor (0.8) in Nepal. The non-specific register column in Pokhara, Nepal substantially underestimated coverage (20.7%).

**Conclusions:**

Exit survey-report highly underestimated observed CHX coverage in all three hospitals. Routine register-recorded coverage was closer to observer-assessed coverage than survey reports in all hospitals, including for caesarean births, and was more accurately captured in hospitals with a specific register column. Inclusion of CHX cord care into registers, and tallied into health management information system platforms, is justified in countries with national policies for facility-based use, but requires implementation research to assess register design and data flow within health information systems.

**Supplementary Information:**

The online version contains supplementary material available at 10.1186/s12884-020-03338-4.

## Key findings

**Table:** 

**What is known and what is new about this study?** • Application of 7.1% chlorhexidine digluconate for umbilical cord care (CHX) is recommended by the World Health Organization for home births in high newborn mortality settings, and is being scaled up in many countries, including for hospital births. • There are limited data tracking coverage at national or global levels. Although the Demographic and Health Surveys’ (DHS) additional modules have optional questions, there is little country uptake and these are not yet validated. • EN-BIRTH is the first multi-country observational study to assess validity of the use of CHX measurement (*n* = 12,379 observed newborns) compared to women’s report on exit survey and routine register-recording. **Survey – what did we find and what does it mean?** • We used the same survey questions as the DHS optional newborn module. • We found high observed coverage (96.6%) but also high (71.5%) “don’t know” replies from women reporting on application of CHX to their newborn’s umbilical cord. • Survey-reported coverage (11.3%) vastly underestimated observed coverage (96.6%) in hospitals and was extremely inaccurate. **Register – what did we find and what does it mean?** • Registers designed with a specific column more accurately recorded the high coverage of CHX application than those with non-specific columns. • The same register design performed differently in two separate facilities, and CHX coverage was slightly overestimated (9.0%) in one. • Qualitative data highlighted opportunities to improve register design, completion and use, especially training and supervision. **Gap analysis for quality of care and measurement** • Almost all newborns observed received CHX, hence the coverage gap was small, except after caesarean birth in one facility. • Quality of care in terms of timing revealed that most newborns (92.2%) received CHX within 1 h of birth. • Further research is needed to assess the optimal sequencing of immediate newborn care interventions to avoid separation of women and newborns, promote early breastfeeding, and ensure that CHX application enhances and does not delay time sensitive practices. **What next and research gaps?** • CHX has become a part of immediate newborn care policy in many countries, including for facility births. • For institutional births, well-designed routine registers have higher accuracy than women’s exit survey-reports, but research is required on design and data flow in health management systems. • Given the poor performance of survey-reported data for facility-based CHX use, further survey validation research should focus on home births, or postnatal application by women to explore how best to measure coverage outside facility-based systems.	

## Background

Globally, almost half of under-five mortality occurs during the first 4 weeks after birth, the neonatal period [1, 2]. Infection is a leading cause of neonatal mortality, particularly in high-mortality contexts in low- and middle-income countries [3, 4]. The newborn umbilical stump is an important entry point for sepsis and systemic infections [5, 6]. Research has shown that the application of 7.1% chlorhexidine digluconate (CHX), a broad-spectrum antiseptic, to the umbilical cord can reduce mortality, especially if applied on the first day of life as per World Health Organization (WHO) guidelines [7]. The highest gain is for very low birthweight neonates, where a dose response by birthweight is evident, and newborns benefit from early application [8–10]. Beyond day 1, CHX application reduces the risk of local infection to the cord stump (from 56 to 27%) and may also reduce later mortality risk [11]. Hence this low-cost intervention could contribute to reducing the burden of mortality due to neonatal sepsis in the first week of life [8, 12–14].

The WHO recommends clean, dry cord care for all newborns and daily CHX application to the umbilical stump for the first week of life for home births in high neonatal mortality settings (> 30 deaths/1000 live births) [6, 15]. These recommendations reflect the evidence available at the time, which included randomised trials mainly conducted in high-mortality home birth settings in south Asia, including Nepal and Bangladesh [6]. These guidelines noted the potential for CHX application to lower or replace traditional practices, including application of harmful substances such as cow dung [6]. There are now two studies in Africa of umbilical cord cleansing for home births, but these did not report significant mortality benefits [16, 17].

Despite many concerns regarding hospital acquired infections [18, 19], no randomised trial has rigorously assessed mortality effect for facility births to date, although there is an ongoing randomised controlled trial testing a single application of 4% chlorhexidine in Uganda [20]. Analysis from 3223 facility births in Bangladesh and Nepal observed significant decreases in mortality in newborns who received CHX [21]. At least 15 countries have implemented a national policy for use of CHX; most, including Bangladesh and Nepal, have a national policy for universal CHX coverage for all births, including those in facilities [22].

Tracking coverage of high impact evidence-based interventions is needed to drive progress to achieve Sustainable Development Goal 3.2, ending preventable neonatal mortality. Currently, umbilical cord care coverage is measured by population-based household survey programmes such as the Demographic and Health Surveys (DHS) Program and Multiple Indicator Cluster Surveys (MICS), typically conducted every 2–5 years (Additional file 1). MICS includes a standard question on cord care practices [23]; however, in DHS this is included in an optional add-on newborn care module with the question: “Was chlorhexidine applied to the stump at any time?” [24] (Additional file 1). Household surveys have many strengths, including a nationally representative sample. However, previous validity research findings for indicators of practices and interventions around the time of birth are mixed. At a minimum, women can only report on clinical interventions they have either discussed with health providers, directly experienced during a state of regular consciousness, or have witnessed [25–30]. Only one previous research study has tested validity of survey CHX measurement in Nigeria, although this had a small sample size [25].

Where CHX application is implemented in facilities, the opportunity exists to track coverage using facility register data for routine health management information systems (HMIS). These data have the advantage of being aggregated and available for use in decision making on a far more frequent basis than household survey data, and thus have the potential to regularly inform quality improvement efforts at subnational levels of the health system. Data accuracy must be trusted to promote use for planning, management, resource allocation and quality monitoring [31]. No previous research has assessed validity of register-recorded measures for CHX coverage [7].

The *Every Newborn* Action Plan*,* supported by all United Nations member states and > 80 development partners, includes an ambitious measurement improvement roadmap [32, 33] with an urgent focus on validating indicators for care and outcomes around the time of birth. As part of this roadmap, the *Every Newborn*– Birth Indicators Research Tracking in Hospitals (EN-BIRTH) study was a mixed-methods observational study of > 23,000 hospital births in three countries – Bangladesh (BD), Nepal (NP) and Tanzania – and aimed to validate selected newborn and maternal indicators for routine facility-based tracking of coverage, quality of care, and outcomes [34, 35]. At the time of study design Tanzania did not have a policy for CHX; therefore, this paper focuses on Bangladesh and Nepal.

## Objectives

This paper is part of a supplement based on the EN-BIRTH multi-country validation study, *‘Informing measurement of coverage and quality of maternal and newborn care’*, and focuses on application of CHX, with three main objectives:

1. **Assess NUMERATOR accuracy/validity** of measurement for a coverage indicator of single application 7.1% chlorhexidine to the umbilical cord stump via exit-survey of women’s report and routine labour ward register data, compared to observation (gold standard).
2. **Analyse GAPS in coverage and quality of care, and measurement for application of 7.1% CHX to the umbilical cord stump**, including observation data to assess right time, right substance applied and experience of care (assessed via survey-report regarding recall of communication of care).
3. **Evaluate BARRIERS AND ENABLERS to routine labour ward register-recording** for CHX through qualitative interviews regarding register design, completion and use.

## Methods

**Fig. 1:**
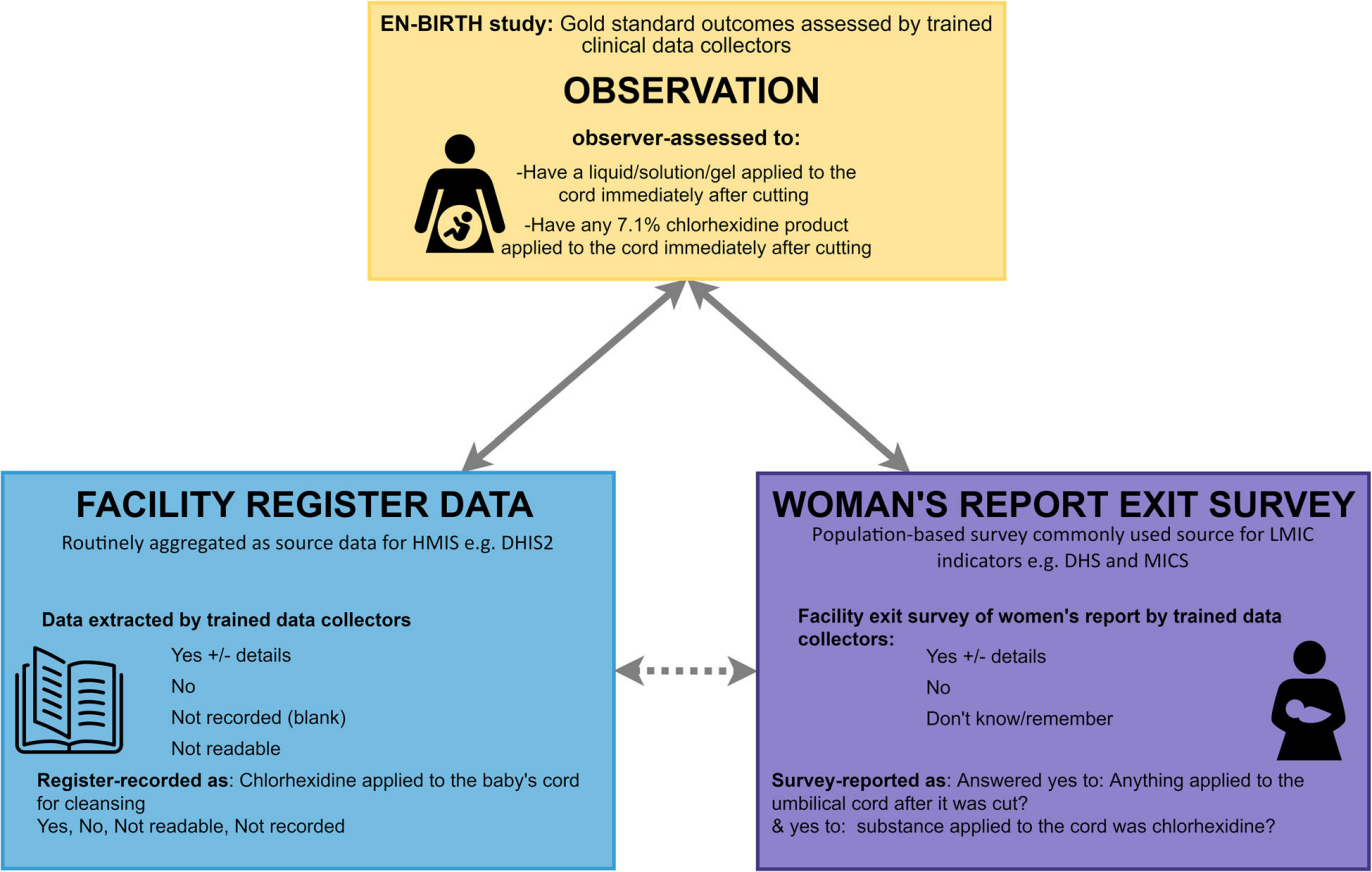
Chlorhexidine validation design, EN-BIRTH study. EN-BIRTH: *Every Newborn* Birth Indicators Research Tracking in Hospitals; HMIS: Health Management Information Systems*;* DHIS2: District Health Information Software 2; DHS: Demographic and Health Surveys; MICS: Multiple Indicator Cluster Surveys. 7.1% Chlorhexidine solution applied to the umbilicus

EN-BIRTH was an observational mixed-methods study and compared data from clinical observers about CHX application (gold standard) to women’s exit-interview survey reported coverage (Additional file 2) and routine register-recorded coverage (Fig. 1). Trained health workers observed participants 24 h per day throughout the woman’s admission to labour and delivery ward. They recorded data on care and outcomes, including application of CHX to the umbilical cord stump (Fig. 1). All data collectors were given training to recognise the correct product for local use. Data were collected using a custom-built android tablet-based software application that included timestamps for observation data (July 2017–July 2018) in three public hospitals providing comprehensive emergency obstetric and newborn care (CEmONC) and application of CHX: Maternal and Child Health Training Institute (MCHTI), Azimpur and Kushtia General Hospital in Bangladesh, and Pokhara Academy of Health Sciences in Nepal (Additional file 3). Participants were consenting women admitted in labour in the three study sites (Additional file 4). Metadata definitions for the CHX indicator are also shown (Additional file 1). All statistical analyses were undertaken using Stata 15.0 (Stata Corporation, College Station, TX, USA). Results were reported in accordance with STROBE statement checklists for cross-sectional studies (Additional file 5). Detailed information regarding the research protocol, methods, and analysis has been published separately [34, 35].

### Labour ward registers

All three study hospitals used pre-printed routine labour ward registers. The register design in Bangladesh changed to a standardised national labour ward register during the EN-BIRTH study. The revised Bangladesh register had a new specific column for documenting CHX application labelled, *7.1% Chlorohexidine used on the umbilical cord.* A blank box was provided where staff were instructed to tick for ‘given’ and leave blank for ‘not given’. In Pokhara NP, CHX application was recorded in a non-specific column labelled “general remarks” and health workers were instructed to document ‘CHX is given’ or leave blank if ‘not given’. Only results from revised registers for the Bangladesh sites are presented in this paper.

### Objective 1: Numerator validation

We compared exit survey-reported and register-recorded coverage to observer-assessed coverage of CHX and stratified by hospital and mode of birth: vaginal births and caesarean births. Percentages of “don’t know” replies for exit survey questions, and ‘not recorded or not readable’ for register-recorded data were also calculated. In line with how DHS/MICS typically analyse ‘yes/no/don’t know’ questions, we compared survey-reported results with “don’t know” considered as “no” against “don’t know” excluded. Similarly, for register-recorded coverage, we compared results with “not recorded” considered as “no” and also excluded.

We calculated absolute differences between measured coverage (survey or register) and observed coverage of CHX use to understand under- or over-estimation at the population level. Using two-way tables, we calculated individual-level validity statistics: sensitivity, specificity, and percent agreement ((true positive + true negative)/total) of register-recorded and survey-reported CHX coverage to observed coverage. Area under the curve, inflation factor, positive predictive value, and negative predictive value were also calculated. We report results where column totals were > 10 in the two-by-two tables. Pooled results for validity analyses were calculated using random effects meta-analysis, presented with I^2^, τ^2^, and heterogeneity statistic (Q). We calculated “validity ratios” (against gold standard), heat-mapping results using standard data quality review cut-offs (over/underestimate by 0 – 5%, by 6–10%, by 11–15%, by 16–20% and > 20%) [36]. All calculations included 95% confidence intervals (CI) where appropriate.

### Objective 2: Gap analysis for coverage and quality of care, and measurement

We analysed four gaps for CHX use in hospitals: 1) Coverage gap between the target population (all live births) and the observed coverage of CHX. 2) Quality of care gap for content - between those newborns observed to have *anything* applied to the cord and those correctly having CHX applied. Current WHO guidelines suggest CHX application within the first day, however ‘correct’ time was taken to be within 1 h of birth, because observations were restricted to the labour and delivery ward in this study. 3) Measurement gap for register records (observed and register-recorded coverage gap). 4) Measurement gap for survey reports (observed and survey-reported coverage of any cord cleansing after birth).

### Objective 3: Barriers and enablers to routine recording

As part of the EN-BIRTH study, qualitative interviews were conducted to understand the barriers and enablers for routine register-recording of interventions around birth. Qualitative data were collected from a purposive sample of health workers (nurses, midwives and doctors) and EN-BIRTH study data collectors. Interviews were recorded, transcribed, translated, and NVivo (QSR International Pty Ltd. Version 12) software was used for data management.

Detailed qualitative methods and overall results are available in an associated paper [37]. Qualitative analysis began with identifying emerging themes based on the Performance of Routine Information System Management (PRISM) conceptual framework [38]. This paper specifically presents themes relating to the recording of umbilical application of CHX.

## Results

### Sample description and selection

**Fig. 2:**
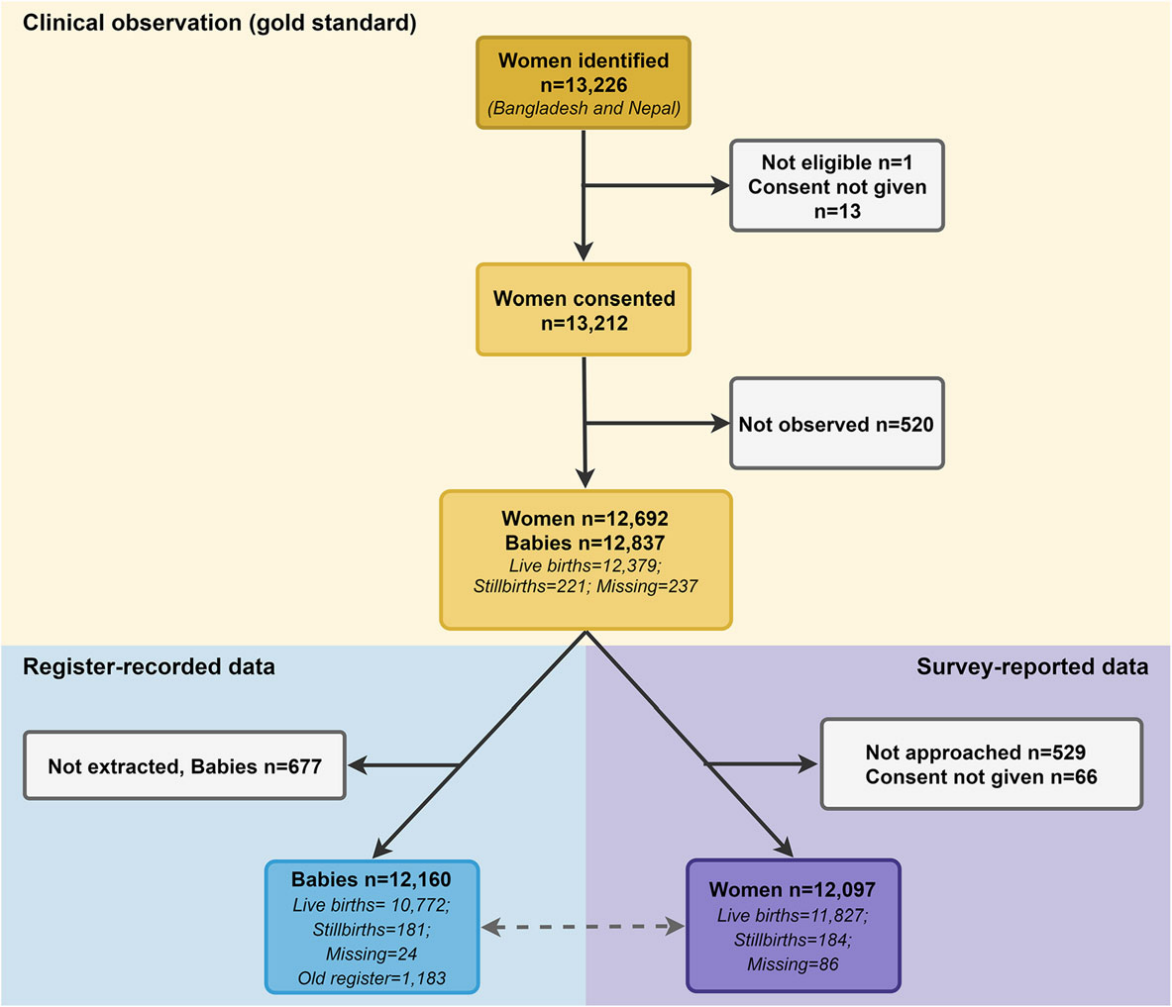
Flow diagram for cord Chlorhexidine application in Bangladesh and Nepal, EN-BIRTH study. 7.1% Chlorhexidine solution applied to the umbilicus

Among 12,379 live births observed for CHX use on labour wards in Bangladesh and Nepal, 10,772 live births (87.0%) were included for register extraction (Fig. 2). 95.3% of women completed an exit survey (12,097 women interviewed out of the possible 12,692 women observed) which correspond to 95.5% live births (11,827 live births out of the possible 12,379 live births observed).

Birth outcomes and background characteristics are shown in Table 1. Almost three-quarters (72.8%) of births in Azimpur BD were via caesarean section compared to 40.3% in Kushtia BD and 15.5% in Nepal. Overall, more than 60% of the women were aged between 20 and 29 years, and 2.7% were < 18 years. Completion of secondary education was lowest in Kushtia BD (36.1%) and highest in Pokhara NP (61.2%). Approximately 13.4% of newborns were < 2500 grammes across the three facilities.

**Table 1:** Characteristics of women observed in labour and delivery wards, EN-BIRTH study (*n* = 12,837)

	Bangladesh	Bangladesh	Nepal	All sites
	Azimpur Tertiary	Kushtia District	Pokhara Regional	
	n (%)	n (%)	n (%)	n (%)
**A). Total newborns who were observed (Denominator)**	2936	2459	7442	12837
**Birth outcome - Live Birth**	2896 (98·6)	2308 (93·9)	7175 (96·4)	12379 (96.4)
**Newborn condition at L&D discharge**				
Alive	2895 (98.6)	2302 (93.6)	7171 (96.4)	12368 (96.3)
Stillbirths	11 (0.4)	74 (3)	126 (1.7)	211 (1.6)
Neonatal death	1 (0)	6 (0.2)	4 (0.1)	11 (0.1)
Baby not delivered	2 (0.1)	2 (0.1)	6 (0.1)	10 (0.1)
Birth outcome not observed	27 (0.9)	75 (3.1)	135 (1.8)	237 (1.8)
**Mode of birth**				
Normal vaginal birth	767 (26.4)	1364 (56.6)	5840 (79.2)	7971 (62.8)
Vaginal breech/ Vacuum/ Forceps	1 (0)	0 (0)	349 (4.7)	350 (2.8)
Caesarean Section	2119 (72.8)	972 (40.3)	1140 (15.5)	4231 (33.3)
Not observed	23 (0.8)	76 (3.2)	41 (0.6)	140 (1.1)
**Birthweight of baby <2500 g**	353 (11.9)	473 (19.3)	897 (12.1)	1723 (13.4)
**Sex Female/Girl baby**	1427 (49)	1128 (46.8)	3335 (45.3)	5890 (46.4)
**B). Total women who were observed**	2910	2412	7370	12692
**Women’s Age**^a^				
<18 years	25 (0.9)	3 (0.1)	311 (4.2)	339 (2.7)
18-19 years	475 (16.3)	197 (8.2)	817 (11.1)	1489 (11.7)
20-24 years	1158 (39.8)	954 (39.6)	3080 (41.8)	5192 (40.9)
25-29 years	867 (29.8)	736 (30.5)	2114 (28.7)	3717 (29.3)
30-34 years	297 (10.2)	373 (15.5)	827 (11.2)	1497 (11.8)
35+ years	88 (3)	149 (6.2)	221 (3)	458 (3.6)
Mean (SD)	23.9 (4.5)	24.9 (4.9)	24.2 (4.7)	24.3 (4.7)
**Women’s education**^a^				
No education	39 (1.3)	77 (3.2)	268 (3.6)	384 (3)
Primary incomplete	111 (3.8)	127 (5.3)	252 (3.4)	490 (3.9)
Primary complete	339 (11.6)	347 (14.4)	302 (4.1)	988 (7.8)
Secondary incomplete	985 (33.8)	954 (39.6)	1637 (22.2)	3576 (28.2)
Secondary complete or higher	1273 (43.7)	870 (36.1)	4509 (61.2)	6652 (52.4)
Missing	163 (5.6)	37 (1.5)	402 (5.5)	602 (4.7)
Mean (SD)	8.8 (4.1)	8.2 (3.6)	9.6 (4.4)	9.1 (4.2)

^a^Data were collected from women’s registration and survey report

### Objective 1: Numerator validation

**Fig. 3:**
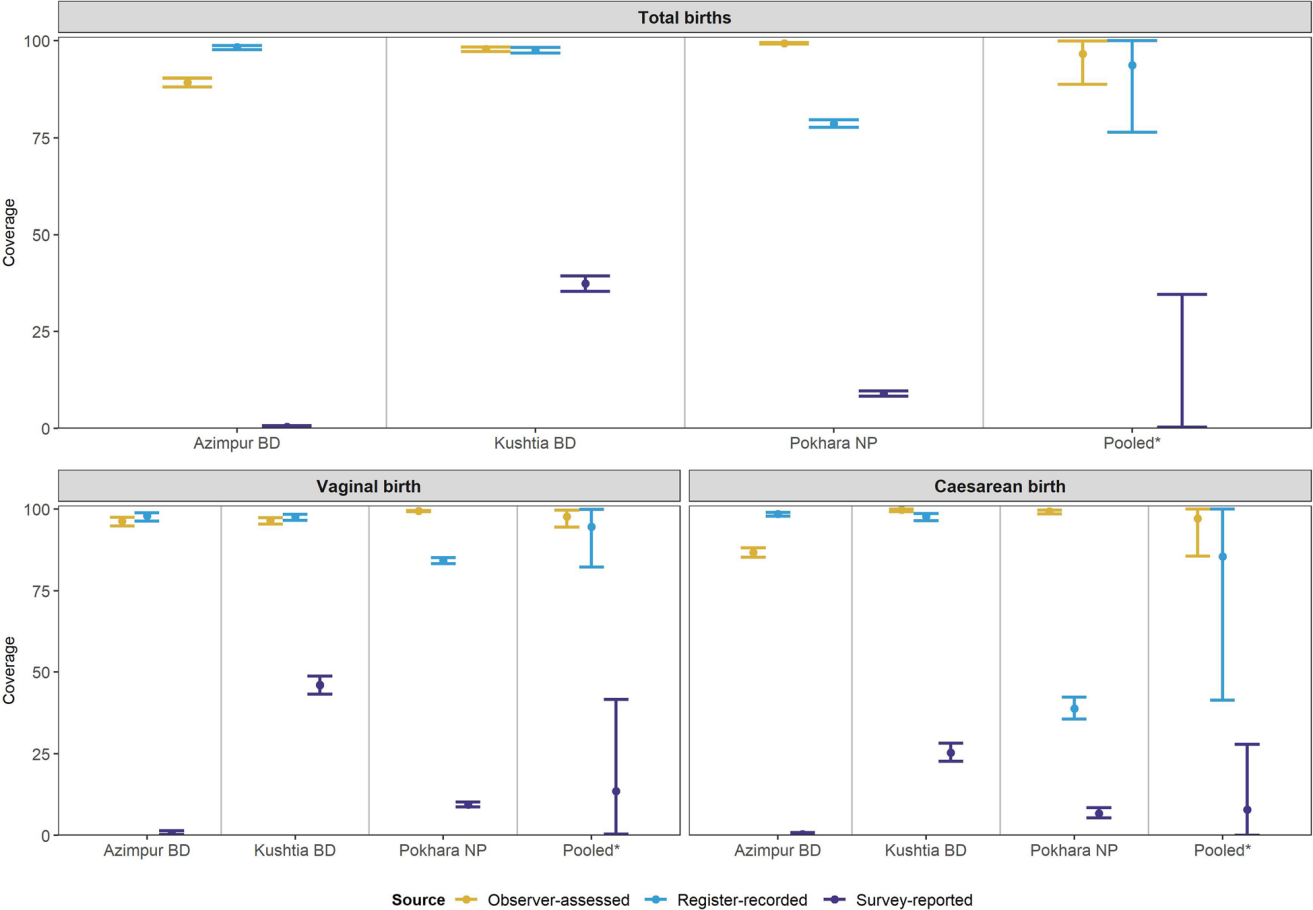
Coverage rates for Chlorhexidine cord application measured by observation, register and exit-survey, EN-BIRTH study (*n* = 12,379). Register-recorded (*n* = 11,002 live births) and exit survey-reported (*n* = 11,827 live births), split by three hospitals. BD: Bangladesh; NP: Nepal. *7.1%* Chlorhexidine solution applied to the umbilicus

To calculate coverage we used the recommend denominator of all live births. In this analyses we included the following denominators: observer-assessed (*n* = 12,379), register-recorded (*n* = 11,002), and exit survey-reported (n = 11,827) live births. Observer-assessed coverage of CHX application within 1 h of birth was high in all three hospitals for both vaginal births (97.7%, 95% CI 94.4–99.6%) and caesarean sections (97.1%, 95% CI 94.4–99.6%) (Fig. 3).

#### Exit-interview survey-reported validation

CHX coverage was consistently underestimated by survey compared with gold standard in all three sites for vaginal births and caesarean births (Fig. 3). Responses yielded high “don’t know” replies for both vaginal births and caesarean section (68.5%, 95% CI 47.9–85.9% / 76.4%, 95% CI 66.6–85.0%, respectively). Percent agreement was low (18.1%, 95% CI 5.5–35.9%), and analysis criteria (> 10 column count) was only met for one hospital (Table 2). Survey-reported timing of CHX (within 1 h of birth) showed high specificity (94.7% 95% CI 74.3–100.0%) but low sensitivity (6.7% 95%CI 0.0–23.9%) in all hospitals (Additional file 6), including “don’t knows”. Most women (56.1% in Kushtia BD to 79.4% in Pokhara NP) reported that the health worker did not inform them or they did not know if anything was applied to their newborn's umbilical cord (Additional file 7).

**Table 2:** Individual-level validation in exit survey report of Chlorhexidine cord-application, EN-BIRTH study (*n* = 6748)

	Azimpur (BD) Tertiary		Kushtia (BD) District		Pokhara (NP) Regional		Pooled (Random effects)	
	N (%)	(CI)	N (%)	(CI)	N (%)	(CI)	N%	(CI)
Exit-survey denominator	2826 live births		2253 live births		6748 live births			
All modes of birth combined								
Observer prevalence %	2582 (89.3)		2257 (97.9)		7112 (99.4)		96.6	(88.8, 99.9)
Survey-reported prevalence %	12 (0.4)		840 (37.3)		604 (9)		11..3	(0.3, 34.6)
Don’t know responses %	2189 (77.5)		1251 (55.5)		5355 (79.4)		71.5	(57.3, 83.7)
INCLUDES DON’T KNOW AS NO								
10 Cell Counts	No		Yes		No			
% agreement	11.0		38.4		9.5		18.1	(5.5, 35.9)
Sensitivity	**	**	37.7	(35.6, 39.7)	**	**	11.5	(0.3, 34.9)
Specificity	**	**	71.7	(56.5, 84)	**	**	93.0	(66.0, 100.0)
EXCLUDES DON’T KNOW								
> 10 Cell Counts	No		No		No			
% agreement	11.5		83.7		44.2		45.8	(10.0, 84.3)
Sensitivity	2.1	(1.1, 3.6)	84.7	(82.3, 86.9)	44.1	(41.4, 46.8)	39.7	(2.7, 86.7)
Specificity	100	(94.1, 100)	23.5	(6.8, 49.9)	60	(14.7, 94.7)	70	(3.2, 100)
Vaginal births								
Observer prevalence %	731 (96.3)		1290 (96.5)		6075 (99.4)		97.7	(94.4, 99.6)
Survey-reported prevalence %	4 (0.5)		601 (45.9)		536 (9.3)		13.6	(0.3, 41.7)
Don’t know responses %	565 (76.5)		629 (48.1)		4508 (78.6)		68.5	(47.9, 85.9)
INCLUDES DON’T KNOW AS NO								
> 10 Cell Counts	No		Yes		No			
% agreement	4.2		47.8		9.9		17.4	(1.6, 4.4)
Sensitivity	**	**	46.9	(44.1, 49.7)	**	**	13.9	(0.3, 42.5)
Specificity	**	**	72.7	(57.2, 85)	**	**	91.6	(70.3, 100.0)
EXCLUDES DON’T KNOW								
> 10 Cell Counts	No		No		No			
% agreement	5.2	(2.4, 9.6)	88.2	(85.6, 90.6)	44.4	(41.6, 47.3)	44.4	(7.5, 85.6)
Sensitivity	2.4	(0.7, 6)	89.8	(87.2, 92)	44.3	(41.5, 47.2)	42.8	(5, 86.8)
Specificity	100	(47.8, 100)	25	(7.3, 52.4)	60	(14.7, 94.7)	63.2	(11.5, 100)
Caesarean births								
Observer prevalence %	1850 (86.8)		967 (99.8)		1037 (99.2)		97.1	(85.6, 100.0)
Survey-reported prevalence %	8 (0.4)		239 (25.3)		66 (6.7)		7.9	(0.0, 27.9)
Don’t know responses %	1624 (77.9)		622 (65.9)		823 (83.7)		76.4	(66.6, 85.0)
INCLUDES DON’T KNOW AS NO								
> 10 Cell Counts	No		No		No			
% agreement	13.4		25.4		7.1		**	**
Sensitivity	**	**	**	**	**	**	**	**
Specificity	**	**	**	**	**	**	**	**
EXCLUDES DON’T KNOW								
> 10 Cell counts	No		No		No			
% agreement	13.9	(10.9, 17.4)	74.1	(69, 78.8)	42.3	(34, 50.8)	42.4	(7.4, 82.7)
Sensitivity	2	(0.9, 3.9)	74.4	(69.2, 79.1)	42.3	(34, 50.8)	34.7	(0, 88.4)
Specificity	100	(93.6, 100)	0	(0, 97.5)	–	–	34.7	(0, 88.4)

*n* = 12,379 observed live births, *n* = 11,827 live births with survey

** = result suppressed due to 10 or fewer count per column of two-by-two table

7.1% Chlorhexidine solution applied to the umbilicus

#### Register-recorded validation

**Fig. 4:**
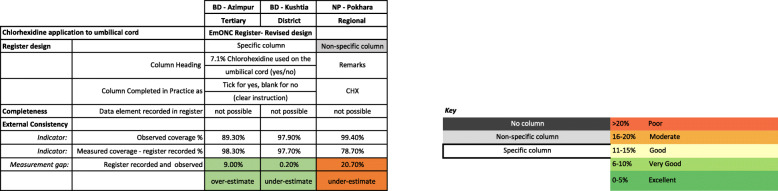
Facility register design and completion approaches for Chlorhexidine application by site, EN-BIRTH study (*n* = 12,379). *n* = 12,379 observed live births, *n* = 10,772 register extracted live births. BD: Bangladesh; NP: Nepal. 7.1% Chlorhexidine solution applied to the umbilicus. In Bangladesh, the registers were revised to a standardised national EmONC register (Additional file 3), neither original facility register had any column for CHX documentation. Completeness calculations were “not possible” for Bangladesh registers as in this design, left blank also meant that the intervention/practice was not done. Reference: Cut-off ranges adapted from WHO Data Quality Review, Module 2 “Desk review of data quality” [36]

Register-recorded CHX application coverage was variable between the three hospital registers. Most accurate was the register-recorded coverage in Kushtia BD, underestimating by only 0.2% (Fig 4). This identical register captured CHX in a specific column and overestimated coverage by 9.0% in Azimpur BD. The least accurate register-recorded coverage was from the non-specific column in Pokhara NP, underestimating coverage by 20.7%. Register performance to measure CHX application was consistently better for vaginal than caesarean births (Table 3). In Pokhara NP, register-recorded coverage was underestimated by 15.1% for vaginal births (99.4–84.3%) and 60.2% for caesareans (99.2–39.0%). Percent agreement was high especially for vaginal births (83.9%) and increased when “don’t know” responses were excluded (98.9%), although all facilities had a column count < 10 (Additional file 8). In Bangladesh, register instructions dictated that the column was left blank when CHX was not applied, which was problematic for analysis because there was no true measure of ‘not given’.

**Table 3:** Individual-level validation of register recording for Chlorhexidine cord-application, EN-BIRTH study (*n* = 6711)

	Azimpur (BD) Tertiary		Kushtia (BD) District		Pokhara (NP) Regional		Pooled (Random effects)	
	N (%)	(CI)	N (%)	(CI)	N (%)	(CI)	N (%)	(CI)
Register-recorded denominator	2222 live births		1839 live births		6711 live births			
All modes of birth combined								
Observer prevalence %	2582 (89.3)		2257 (97.9)		7112 (99.4)		99.6	(88.8, 99.9)
Register-recorded prevalence %	2185 (98.3)		1796 (97.7)		5282 (78.7)		93.7	(76.4, 100.0)
Not recorded	13 (0.6)		41 (2.2)		1394 (20.8)		5.4	(0.0, 23.5)
Not readable	0 (0)		0 (0)		4 (0.1)		0.0	(0.0, 0.1)
INCLUDES NOT RECORDED AND NOT READABLE AS NO								
> 10 Cell counts	No		No		Yes			
% agreement	88.6		96.4		78.7		89.0	(76.4, 97.1)
Sensitivity	**	**	**	**	79	(78, 80)	93.8	(76.7, 100.0)
Specificity	**	**	**	**	25	(12.7, 41.2)	8.8	(0.0, 28.0)
EXCLUDES NOT RECORDED AND NOT READABLE								
> 10 Cell counts	No		No		No			
% agreement	88.6		96.4		98.9		95.5	(87.7, 99.5)
Sensitivity	**	**	**	**	**	**	**	**
Specificity	**	**	**	**	**	**	**	**
Vaginal births								
Observer prevalence %	731 (96.3)		1290 (96.5)		6075 (99.4)		97.7	(94.4, 99.6)
Register-recorded prevalence %	547 (97.9)		1073 (97.6)		4963 (84.3)		94.5	(82.2, 99.9)
Not recorded	7 (1.3)		25 (2.3)		894 (15.2)		4.9	(0.0, 17.1)
Not readable	0 (0)		0 (0)		3 (0.1)		0.0	(0.0, 0.1)
INCLUDES NOT RECORDED AND NOT READABLE AS NO								
> 10 Cell counts	No		No		No			
% agreement	94.4		95.4		83.9		91.9	(82.1, 98.0)
Sensitivity	**	**	**	**	**	**	**	**
Specificity	**	**	**	**	**	**	**	**
EXCLUDES NOT RECORDED AND NOT READABLE								
> 10 Cell counts	No		No		No			
% agreement	94.4		95.4		98.9		96.6	(92.5, 99.1)
Sensitivity	**	**	**	**	**	**	**	**
Specificity	**	**	**	**	**	**	**	**
Caesarean births								
Observer prevalence %	1850 (86.8)		967 (99.8)		1037 (99.2)		97.1	(85.6, 100.0)
Register-recorded prevalence %	1638 (98.5)		723 (97.7)		318 (39)		85.5	(41.5, 100.0)
Not recorded	6 (0.4)		16 (2.2)		495 (60.7)		12.9	(0.0, 58.9)
Not readable	0 (0)		0 (0)		1 (0.1)		0.0	(0.0, 0.1)
INCLUDES NOT RECORDED AND NOT READABLE AS NO								
> 10 Cell Counts	No		No		No			
% agreement	86.6		**		40.2		79.5	(43.2, 99.3)
Sensitivity	**	**	**	**	**	**	**	**
Specificity	**	**	**	**	**	**	**	**
EXCLUDES NOT RECORDED AND NOT READABLE								
> 10 Cell counts	No		No		No			
% agreement	86.6		**		99.1		95.7	(85.5, 100.0)
Sensitivity	**	**	**	**	**	**	**	**
Specificity	**	**	**	**	**	**	**	**

*n* = 12,379 observed live births, *n* = 10,772 live births with register records

** = result suppressed due to 10 or fewer count per column of two-by-two table

7.1% Chlorhexidine solution applied to the umbilicus

**Fig. 5:**
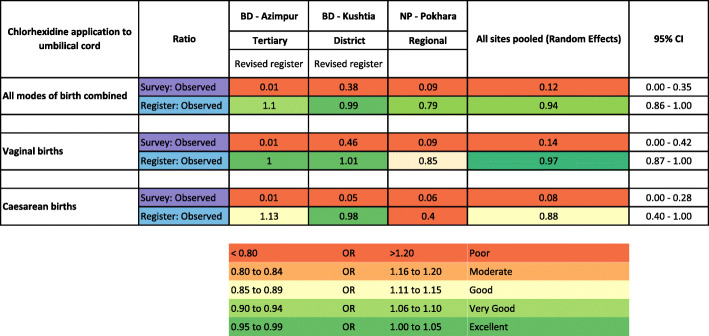
Heat map of validity ratios for chlorhexidine cord application, EN-BIRTH study. BD: Bangladesh; NP: Nepal. Using cut off ranges adapted from WHO Data Quality Review, Module 2 “Desk review of data quality” [36]. Survey-reported to observed and register-recorded to observed. Observation *n* = 12,379 live births, register-recorded *n* = 10,002 live births and exit survey-reported *n* = 11,827 women with live births. 7.1% Chlorhexidine solution applied to the umbilicus

Comparison of heat-mapped validity ratios for exit-survey or register-recorded measures compared with observer-assessed suggested that register data for CHX was more accurate (ratio 0.94) than women’s report (ratio 0.12) Fig 5. It was categorised as ‘good’ for vaginal births and caesareans (ratios ~ 1.00) in both Bangladesh hospitals. Vaginal births were ‘moderate’ (ratio 0.85) and caesareans ‘poor’ (ratio 0.40) in Nepal. Validity ratios for survey-reported results were categorised as ‘poor’ (ratio range 0.01 to 0.38) in all facilities (Fig. 5).

### Objective 2: Gap analysis for coverage, quality of care, and measurement

**Fig. 6:**
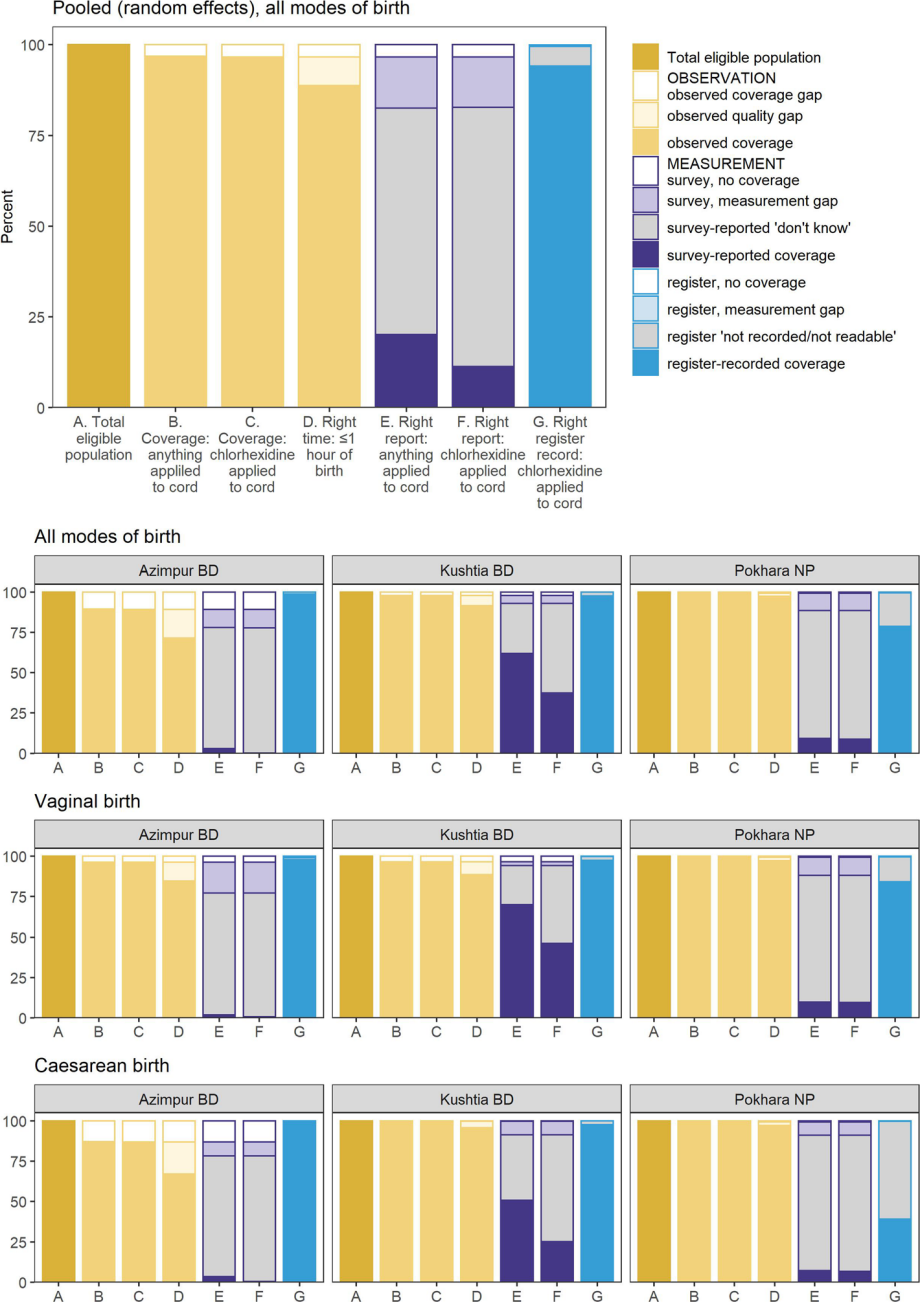
Gap analysis for Chlorhexidine cord application coverage and quality, EN-BIRTH study (*n* = 12,379). Register-records *n* = 11,002 live births, and exit survey-report *n* = 11,827 women with live births. ‘Right time’ < 1 h was used here as the observation period is only during admission to labour and delivery wards. The current WHO recommendations advise that Chlorhexidine application should be completed within the first week of life [6]. 7.1% Chlorhexidine solution applied to the umbilicus

Almost all newborns in these facilities were observed to receive CHX. The coverage gap was very small for the target population of all live births (Fig. 6). Within these facilities, there was close observed alignment between application of anything and CHX to the cord, however this leads to a measurement gap in survey report where women were more able to report that something was applied (17.8%), rather than CHX (12.3%) (Additional file 9). Quality of care gap analysis showed timing distribution (less than 1 h of birth) was similar among each facility and by mode of birth. Survey reported “don’t knows” were higher in Azimpur BD and Pokhara NP considering all modes of birth.

### Objective 3: Barriers and enablers to routine recording

These findings were specific to recording practices for CHX, but more detailed qualitative results are available in a supporting paper [37]. Respondents in all hospitals talked of the complexity of multiple registers (both formal and informal register books) to record interventions around birth, including CHX (Fig. 4).

In Bangladesh the revised register design was an enabler:

“*Previously we did not document the care of chlorhexidine in registers as it did not (have) space to write. Now this new register has a specific column where we can document whether chlorhexidine was applied or not.*” -Health worker, Azimpur BD

Most respondents from Bangladesh and some in Nepal agreed that it is useful to have a specific column on CHX in the register:

“*Now, more information is added to the delivery register than before. For example, information related chlorhexidine was not included before.*”-Health worker, Kushtia BD

In Bangladesh, respondents from Kushtia reported that they were not confident to record in the new register due to a lack of formal training. This was in contrast to Azimpur, where more formal supervision and training was provided during the rollout of revised national registers:

“*We haven’t received any formal training from the hospital. The in-charge has told us verbally how to fill up the register and write information in other informal books.*” -Health worker, Azimpur BD

## Discussion

EN-BIRTH is the largest observational study to assess validity of coverage measurement for CHX application through women’s exit-interview survey to date, and the first to assess validity of routine hospital registers. Our multi-site, multi-country design enabled comparisons between and within countries. The large sample size enabled the first assessment of how caesarean section affects CHX coverage measurement.

For household surveys, CHX coverage questions are already included in the optional newborn module of DHS. Our data collectors also showed a visual prompt (a picture of a CHX bottle) to the mother, in line with survey procedures used by DHS for this question. Survey-reported validation results showed substantial underestimation of coverage, especially after caesarean section. “Don’t know” responses exceeded 50% regarding if *any* substance, or CHX specifically, was applied to the cord. These findings are consistent with other research that shows low accuracy of survey-report for clinical interventions around the time of birth [25, 28–30].

A recent study from Nigeria showed much lower “don’t know” replies (5%), and high sensitivity and specificity [25]. Nigeria uses a multi-day regimen in contrast to Bangladesh and Nepal, where a single application is the national standard. In settings using the multi-day approach, families are responsible for continuing daily CHX as part of cord care, and it is therefore an imperative that they receive information and training on how to do this. Using our time-stamped data, we learned that CHX was applied very quickly after birth (median time 2–4 min), so it is likely that the mother was not aware of the multi-step process of clamping, tying, cutting the cord, and applying CHX. In the Nigerian study, it is possible that CHX application was outside the immediate postpartum period, perhaps later during the first day (or days after birth). The context of this study was in primary health care facilities, in contrast to our study in busy CEmONC hospitals. Women could have experienced less separation from their newborn and thus been able to see the CHX applied to the cord, or indeed may have had to buy the CHX, or apply it personally. Alternatively, the variation between findings may be associated with the quality of health worker communication to women. Exit survey findings suggest that health worker communication needs improvement. Only 0.1–5.6% of women reported that health workers told them why CHX was used. This lack of awareness could be driven by the proximity of events to birth, or a communication failure between health workers and women.

The register data underestimated coverage in two hospitals, performing poorly in one out of three. Register design was found to be an important factor in the accuracy of register-recorded coverage in this study; registers with specific columns outperformed those with non-specific columns. However, in Bangladesh registers, completion instructions meant it was not possible to understand whether the intervention was deliberately ‘not given’ or was not recorded in the register for other reasons (i.e. forgotten). Global guidance around register design and indicator prioritisation is required, although implementation and supportive supervision are also crucial. Both hospitals in Bangladesh used the same register design and instructions; however, they did not perform equally. This may be related to different implementation strategies, as Azimpur BD staff received more detailed training and ongoing support during register rollout.

To date, validation research for tracking of cord care practices has focused on population-based survey platforms with no published evaluation regarding routine facility-based measurement systems. This is a major gap, given as many as 20 countries have a national policy for CHX that includes facilities, and demonstrates the need for inclusion of CHX as part of the WHO policy portal [22]. To our knowledge, EN-BIRTH is the first study to assess validity of CHX measurement from routine registers. Register design was found to be an important factor in the accuracy of register-recorded coverage in this study, as registers with specific columns outperforming non-specific columns. However, the specific column in Azimpur BD was ticked when CHX was not given and demonstrates the need for consistent implementation, as well as design.

The increasing proportion of caesarean section births worldwide has important implications for both care and measurement. In one hospital in our study, women who had caesarean underestimated CHX coverage by 75%. In the other two sites there was very little difference between vaginal and caesarean births. Newborns may be cared for separately from their mothers after surgery, and caesarean birth may exacerbate communication gaps, especially if the woman had a general anaesthetic or was unwell following surgery.

Interestingly, the high coverage and timely application of CHX is in marked contrast to low coverage for breastfeeding, where we found early initiation in the first hour after birth to be just 10.9% across all five EN-BIRTH study sites [39]. Immediate newborn care is part of essential newborn care and includes a number of practices such as delayed cord clamping, breastfeeding, and skin-to-skin contact, which are needed in the first few minutes after birth. Pre-discharge interventions such as eye care, vitamin K, newborn assessment, cord care and immunisations are required; all should be implemented with a focus on zero separation of women and their newborns [15, 39].

The immediate newborn care practice with the strongest evidence base is early initiation of breastfeeding, with high impact for reducing newborn morbidity and mortality and contributing to health gains for the woman [40–42]. CHX application does not yet have strong evidence regarding facility-based application or for requiring application within minutes. Under time pressure, health workers might prioritise more easily achieved simple tasks, such as CHX application, over potentially time-consuming practices like assisting a mother and baby to breastfeed. Other possibilities of why CHX was prioritised at our study sites might include location of the CHX product (which is only available on the labour ward, rather than postnatal wards) or short admission stays where staff take the opportunity immediately. There are important research questions around the sequencing for immediate and essential newborn care practices to optimise mortality impact, especially with increasing time pressures on the few midwives and other health care professionals.

### Strengths and limitations

Strengths of this study include direct observation as the gold standard, data collection by trained providers using a custom-built tablet application with timestamping, the large sample size and the multi-country, multi-site contexts.

In terms of limitations, we note that validation results are based on CEmONC hospitals, which might not be generalisable to lower levels of care or for women who give birth at home or in private facilities. The presence of researchers could have influenced how health workers completed routine registers (Hawthorne effect) [43]; however, assessment of pre- and during study register data quality is published separately and shows very little difference over time [35].

Our survey questions were aligned to the current DHS optional survey module questions regarding applications to the umbilical cord. However, for EN-BIRTH, we asked women at exit interviews with a short recall period, rather than 2–5 years later, as is usual practice in population-based surveys. Hence, our results could overestimate the validity of measurement for these survey questions, since women may be more likely to accurately report care in this shorter time interval (very soon after birth). Conversely, many women reported “don’t know” and it is possible that for home births they may have known more about what was done to their newborn’s cord.

### Research to improve measurement

Assessment for impact of CHX in facility settings is ongoing, with results from a trial in Uganda expected soon [20]. For countries that already have a policy of facility-based CHX cord application, further implementation research to explore how register design, filling and use can improve data quality is required. Such research should include assessment of health worker training and support. In addition to assessment of data flow and data quality for this indicator’s inclusion in national routine HMIS, evidence of feasibility and cost effectiveness are also required [34]. For home births in high mortality contexts, validation of survey questions regarding women’s report of CHX application on the day of birth and afterwards is necessary. These studies could also explore use of visual prompts as used by DHS, such as a picture of the commodity packaging most commonly used in that context.

## Conclusions

Routine register data performed better than exit survey-report for measurement of CHX coverage in hospitals. Routine registers are a promising source of data where there is a national policy for facility-based CHX application. Further research should assess the opportunity costs in time for health workers to record, as well as utility of the data if coverage is already extremely high. Attention to home births is essential to ensure the poorest and most at-risk families are not omitted from coverage measurement.

## Supplementary Information

**Additional file 1.** Chlorhexidine question wording compared with DHS/MICS. DHS: Demographic and Health Surveys; MICS: Multiple Indicator Cluster Surveys.

**Additional file 2.** EN-BIRTH cord care survey questionnaire used to collect information about cord care and Chlorhexidine cord cleansing.

**Additional file 3.** EN-BIRTH study data collection dates by site and time elapsed between birth and exit survey.

**Additional file 4.** Ethical approval of local institutional review boards for EN-BIRTH study.

**Additional file 5.** STROBE statement.

**Additional file 6.** Individual-level validation in exit-survey report of umbilical cord care practices, EN-BIRTH study (*n* = 12,379).

**Additional file 7.** Exit-survey reported health-worker communication of Chlorhexidine application, EN-BIRTH study (*n* = 11,639 live births).

**Additional file 8.** Validation register-recorded umbilical cord care practices, EN-BIRTH study (*n* = 10,772).

**Additional file 9.** Descriptive data for observer-assessed, register-recorded, and survey-reported Chlorhexidine cord application, EN-BIRTH study (*n* = 12,379 live births).

## Data Availability

The datasets generated during and/or analysed during the current study are available on LSHTM Data Compass repository, https://datacompass.lshtm.ac.uk/955/.

## Abbreviations

BD: Bangladesh
CEmONC: Comprehensive Emergency Obstetric and Newborn Care
CHX: 7.1% Chlorhexidine application to the umbilical cord
CIFF: Children’s Investment Fund Foundation
DHS: The Demographic and Health Surveys Program
EN-BIRTH: *Every Newborn*-Birth Indicators Research Tracking in Hospitals study
HMIS: Health Management Information System
icddr,b: International Centre for Diarrhoeal Disease Research, Bangladesh
LSHTM: London School of Hygiene & Tropical Medicine
MCHTI: Maternal and Child Health Training Institute, Azimpur, Bangladesh
MICS: Multiple Indicator Cluster Survey
NP: Nepal
PRISM: Performance of Routine Information System Management
UNICEF: United Nations International Children’s Emergency Fund
WHO: World Health Organization
