# Supplementary material for: Chlorhexidine for facility-based umbilical cord care: EN-BIRTH multi-country validation study
Source: BMC Pregnancy Childbirth. 2021 Mar 26;21(Suppl 1):239. doi: 10.1186/s12884-020-03338-4 (PMC7995704; doi:10.1186/s12884-020-03338-4)
Supplement: Supplementary file 1 — Additional file 1. Chlorhexidine question wording compared with DHS/MICS. DHS: Demographic and Health Surveys; MICS: Multiple Indicator Cluster Surveys. [file 12884_2020_3338_MOESM1_ESM.pdf]

SUPPLEMENT TITLE:
Every Newborn BIRTH multi-country validation study: informing measurement of coverage and quality of maternal and newborn care

PAPER TITLE:
Chlorhexidine for facility-based umbilical cord care: EN-BIRTH multi-country validation study

Additional File 1: Chlorhexidine question wording compared with DHS/MICS

	**EN-BIRTH**	**DHS-7 Woman's Questionnaire and supplemental modules**	**MICS (Questionnaire for individual women, MICS6)**
Anything applied to cord	After the cord was cut, was anything applied to the stump of the cord at any time?	NB7 (Newborn care supplemental module). Was anything applied to the stump of the cord at any time?	MN30. After the cord was cut and until it fell off, was anything applied to the cord?
Chlorhexidine applied to cord	What was applied to the cord? (Show tube)?<br>PROBE: Anything else?	NB8 (Newborn care supplemental module). What was applied? Anything else?<br>If "chlorhexidine" not reported: CH2. Was chlorhexidine applied to the stump at any time?	MN31. What was applied to the cord?<br>Probe: Anything else?

DHS: Demographic and Health Surveys; MICS: Multiple Indicator Cluster Surveys
