# Supplementary material for: Chlorhexidine for facility-based umbilical cord care: EN-BIRTH multi-country validation study
Source: BMC Pregnancy Childbirth. 2021 Mar 26;21(Suppl 1):239. doi: 10.1186/s12884-020-03338-4 (PMC7995704; doi:10.1186/s12884-020-03338-4)
Supplement: Supplementary file 2 — Additional file 2. EN-BIRTH cord care survey questionnaire used to collect information about cord care and Chlorhexidine cord cleansing. [file 12884_2020_3338_MOESM2_ESM.pdf]

SUPPLEMENT TITLE:
Every Newborn BIRTH multi-country validation study: informing measurement of coverage and quality of maternal and newborn care

PAPER TITLE:
Chlorhexidine for facility-based umbilical cord care: EN-BIRTH multi-country validation study

Additional File 2: EN-BIRTH cord care survey questionnaire used to collect information about cord care and Chlorhexidine cord cleansing

	Questions	Options	Skip to
Q1	After the cord was cut, was anything applied to the stump of the cord at any time?	Yes	If Yes go to Q 28a
		No	If No or Don't Know/Don't Remember, End of Interview
		Don't know/don't remember	
Q2	What was applied to the cord? (Show tube)?<br>PROBE: Anything else?<br>Instructions: Show a tube of chlorhexidine to woman	Chlorhexidine (tube)	If No or Don't Know/Don't Remember, End of Interview
		Other, specify: ___________________	
		Don't know/don't remember	
Q3	How long after the cord was cut was chlorhexidine first applied?	Hours __ __	
		Days __ __	
		Don't know/don't remember	
Q4	If chlorhexidine was applied, who applied the treatment within the last 24hours?	Health worker	
		Myself	
		Family member or friend	
		Other: ________________	
		Don't know/don't remember	
Q5	Were you told the reason why the treatment was being applied to the stump?	Yes	If Yes go to Q 31a
		No	If No or Don't Know/Don't Remember, End of Interview
		Don't know/don't remember	
Q6	What was the reason?	Specify____________________	
		Don't know	
		No	
		Don't know/don't remember	
