# Supplementary material for: Chlorhexidine for facility-based umbilical cord care: EN-BIRTH multi-country validation study
Source: BMC Pregnancy Childbirth. 2021 Mar 26;21(Suppl 1):239. doi: 10.1186/s12884-020-03338-4 (PMC7995704; doi:10.1186/s12884-020-03338-4)
Supplement: Supplementary file 6 — Additional file 6. Individual-level validation in exit-survey report of umbilical cord care practices, EN-BIRTH study (n = 12,379). [file 12884_2020_3338_MOESM6_ESM.pdf]

SUPPLEMENT TITLE:

Every Newborn BIRTH multi-country validation study: informing measurement of coverage and quality of maternal and newborn care

PAPER TITLE:

Chlorhexidine for facility-based umbilical cord care: EN-BIRTH multi-country validation study

Additional File 6: Individual-level validation in exit-survey report of umbilical cord care practices, EN-BIRTH study (n=12,379)

	Azimpur (BD)		Kushtia (BD)		Pokhara (NP)		Overall						
	Tertiary		District		Regional		Pooled Random Effects						
	N(%)	(CI)	N(%)	(CI)	N(%)	(CI)	%	(CI)	Q	df	p-val.	i^2	τ^2
**Exit-Survey Denominator**	2826 livebirths		2253 livebirths		6748 livebirths		11827 livebirths						
**Chlorhexidine Applied to the Cord**													
**All modes of birth Combined**													
Observer Prevalence %	2582 (89.3)		2257 (97.9)		7112 (99.4)		96.6	(88.8,99.9)	528.1	2	<0.001	99.6%	0.074
Survey-Reported Prevalence %	12 (0.4)		840 (37.3)		604 (9)		11..3	(0.3,34.6)	1757.1	2	<0.001	99.8%	0.256
Don't know responses %	2189 (77.5)		1251 (55.5)		5355 (79.4)		71.5	(57.3,83.7)	470.0	2	<0.001	99.5%	0.068
**INCLUDES DON'T KNOW AS NO**													
>10 Cell Counts	No		Yes		No								
% agreement	11.0		38.4		9.5		18.1	(5.5,35.9)	876.5	2	<0.001	99.7%	0.128
Sensitivity	0.5	0.2, 0.8	37.7	35.6, 39.7	9.1	8.4, 9.8	11.5	(0.3,34.9)	1653.7	2	<0.001	99.8%	0.021
Specificity	100	98.8, 100	71.7	56.5, 84	94.3	80.8, 99.3	93.0	(66.1,100.0)	49.8	2	<0.001	95.9%	0.348

Positive Predictive Value %	100	73.5, 100	98.5	97.4, 99.2	99.7	98.8, 100	99.9	(98.8,100.0)	5.5	2	<0.001	63.7%	0.063
Negative Predictive Value %	10.6	73.5, 100	2.4	1.6, 3.3	0.6	0.4, 0.8	3.5	(0.0,12.1)	507.7	2	<0.001	99.6%	0.088
AUC	0.5	0.5, 0.5	0.6	0.5, 0.6	0.5	0.5, 0.6							
Inflation Factor	0.00		0.38		0.09								
**EXCLUDES DON'T KNOW**													
>10 Cell Counts	No		No		No								
% agreement	11.5		83.7		44.2		45.8	(10.0,84.3)	1047.2	2	<0.001	99.8%	0.551
Sensitivity	2.1	1.1, 3.6	84.7	82.3, 86.9	44.1	41.4, 46.8	39.7	2.7, 86.7	1518.0	2	<0.001	99.9%	0.830
Specificity	100	94.1, 100	23.5	6.8, 49.9	60	14.7, 94.7	70	3.2, 100	56.4	2	<0.001	96.5%	1.521
Positive Predictive Value %	100	73.5, 100	98.5	97.4, 99.2	99.7	98.8, 100	99.9	98.8, 100	5.5	2	<0.001	63.7%	0.005
Negative Predictive Value %	9.8	7.6, 12.4	2.6	0.7, 6.6	0.4	0.1, 1.2	3.3	0, 12.5	86.4	2	<0.001	97.7%	0.095
AUC	0.5	0.5, 0.5	0.5	0.4, 0.7	0.5	0.3, 0.8							
Inflation Factor	0.02		0.86		0.44								
**Vaginal Births**													
Observer Prevalence %	731 (96.3)		1290 (96.5)		6075 (99.4)		97.7	(94.4,99.6)	78.4	2	<0.001	97.4%	0.023
Survey-Reported Prevalence %	4 (0.5)		601 (45.9)		536 (9.3)		13.6	(0.3,41.7)	1067.1	2	<0.001	99.8%	0.327
Don't know responses %	565 (76.5)		629 (48.1)		4508 (78.6)		68.5	(47.9,85.9)	450.6	2	<0.001	99.5%	0.138

**INCLUDES DON'T KNOW AS NO**													
>10 Cell Counts	No		Yes		No								
% agreement	4.2		47.8		9.9		17.4	(1.6,4.4)	932.9	2	<0.001	99.7%	0.286
Sensitivity	0.6	0.2, 1.4	46.9	44.1, 49.7	9.4	8.6, 10.2	13.9	(0.3,42.5)	1057.7	2	<0.001	99.81%	0.335
Specificity	100	87.2, 100	72.7	57.2, 85	93.8	79.2, 99.2	91.6	(70.3,100.0)	15.3	2	<0.001	86.9%	0.195
Positive Predictive Value %	100	39.8, 100	98	96.5, 99	99.6	98.7, 100	100	(99.5,100.0)	6.8	2	<0.001	71.0%	0.009
Negative Predictive Value %	3.7	2.4, 5.3	4.6	3.2, 6.4	0.6	0.4, 0.8	2.5	(0.3,6.7)	75.0	2	<0.001	97.3%	0.030
AUC	0.5	0.5, 0.5	0.6	0.5, 0.7	0.5	0.5, 0.6							
Inflation Factor	0.01		0.48		0.09								
**EXCLUDES DON'T KNOW**													
>10 Cell Counts	No		No		No								
% agreement	5.2	2.4, 9.6	88.2	85.6, 90.6	44.4	41.6, 47.3	44.4	7.5, 85.6	700.2	2	<0.001	99.7%	0.630
Sensitivity	2.4	0.7, 6	89.8	87.2, 92	44.3	41.5, 47.2	42.8	5, 86.8	796.9	2	<0.001	99.7%	0.731
Specificity	100	47.8, 100	25	7.3, 52.4	60	14.7, 94.7	63.2	11.5, 100	11.4	2	<0.05	82.4%	0.610
Positive Predictive Value %	100	39.8, 100	98	96.5, 99	99.6	98.7, 100	100	99.5, 100	6.9	2	<0.05	71.0%	0.009
Negative Predictive Value %	3	1, 6.8	5.6	1.6, 13.8	0.4	0.1, 1.3	2.2	0, 6.5	13.0	2	<0.05	84.6%	0.029
AUC	0.5	0.5, 0.5	0.6	0.5, 0.7	0.5	0.3, 0.8							

Inflation Factor	0.02		0.92		0.44								
**Cesarean Births**													
Observer Prevalence %	1850 (86.8)		967 (99.8)		1037 (99.2)		97.1	(85.6,100.0)	379.5	2	<0.001	99.4%	0.147
Survey-Reported Prevalence %	8 (0.4)		239 (25.3)		66 (6.7)		7.9	(0.0,27.9)	568.7	2	<0.001	99.6%	0.230
Don't know responses %	1624 (77.9)		622 (65.9)		823 (83.7)		76.4	(66.6,85.0)	90.5	2	<0.001	97.7%	0.036
**INCLUDES DON'T KNOW AS NO**													
>10 Cell Counts	No		No		No								
% agreement	13.4		25.4		7.1		14.5	(6.7,24.7)	123.4	2	<0.001	98.3%	0.051
Sensitivity	0.4	0.2, 0.9	25.3	22.6, 28.2	6.8	5.2, 8.7	8.0	(0.0,28.1)	526.0	2	<0.001	99.6%	0.231
Specificity	100	98.7, 100	50	1.3, 98.7	100	29.2, 100	99.6	(61.9,100.0)	6.34	2	<0.001	68.4	0.368
Positive Predictive Value %	100	63.1, 100	99.6	97.7, 100	100	94, 100	100	(99.8,100.0)	33.3	2	<0.001	0.0%	0.0
Negative Predictive Value %	13.1	11.7, 14.6	0.1	0, 0.8	0.4	0.1, 1.1	2.5	(0.0,14.6)	349.0	2	<0.001	99.4%	0.167
AUC	0.5	0.5, 0.5	0.4	0, 0.9	0.5	0.1, 1.1							
Inflation Factor	0.00		0.25		0.07								
**EXCLUDES DON'T KNOW**	No		No		No								
>10 Cell Counts													
% agreement	13.9	10.9, 17.4	74.1	69, 78.8	42.3	34, 50.8	42.4	7.4, 82.7	324.6	2	<0.001	99.4%	0.575
Sensitivity	2	0.9, 3.9	74.4	69.2, 79.1	42.3	34, 50.8	34.7	0, 88.4	584.8	2	<0.001	99.7%	1.085
Specificity	100	93.6, 100	**	**	**	**	34.7	0, 88.4	584.8	2	<0.001	99.7%	1.085

Positive Predictive Value %	100	63.1, 100	99.6	97.7, 100	100	94, 100	100	99.8, 100	0.3	2	<0.05	0.0%	<0.001
Negative Predictive Value %	12.4	9.5, 15.8	0	0, 4.4	0	0, 4.4	2.2	0, 14.6	0.3	2	<0.001	95.6%	0.164
AUC	0.5	0.5, 0.5	0.4	0, 1	-	-							
Inflation Factor	0.02		0.75		-								
**Chlorhexidine applied to the Cord within 1 hour of birth**													
**All modes of birth Combined**													
Observer Prevalence %	2314 (80)		2159 (93.6)		7006 (97.9)		91.9	(78.5,99.1)	831.1	2	<0.001	99.7%	0.117
Survey-Reported Prevalence %	1 (0)		588 (26.1)		316 (4.7)		6.6	(0.0,23.7)	1336.3	2	<0.001	99.8%	0.194
Don’t know responses %	2199 (77.8)		1482 (65.8)		5643 (83.6)		76.3	(65.5,85.6)	303.6	2	<0.001	99.3%	0.044
**INCLUDES DON’T KNOW AS NO**													
>10 Cell Counts	No		Yes		No								
% agreement	19.8		29.5		6.5		17.4	(5.5,34.2)	799.9	2	<0.001	99.7%	0.117
Sensitivity	0	0, 0.2	26.4	24.5, 28.3	4.7	4.2, 5.2	6.7	(0.0,23.9)	1184.4	2	<0.001	99.8	0.194
Specificity	100	99.3, 100	76.3	68.3, 83.1	96.9	92.1, 99.1	94.7	(74.3,100.0)	109.1	2	<0.001	98.1%	0.265
Positive Predictive Value %	100	2.5, 100	94.4	92.2, 96.1	98.7	96.7, 99.6	100	(99.4,100.0)	11.8	2	<0.001	83.0%	0.024

Negative Predictive Value %	19.8	18.3, 21.3	6.4	5.3, 7.7	2	1.6, 2.3	7.9	(0.6,22.2)	797.6	2	<0.001	99.7%	0.131
AUC	0.5	0.5, 0.5	0.5	0.5, 0.6	0.5	0.5, 0.5							
Inflation Factor	0.00		0.28		0.05								
**Vaginal Births**													
Observer Prevalence %	668 (88)		1229 (91.9)		5983 (97.9)		93.3	(85.3,98.3)	180.2	2	<0.001	98.8%	0.056
Survey-Reported Prevalence %	0 (0)		402 (30.7)		288 (5)		7.5	(0.0,27.4)	760.1	2	<0.001	99.7%	0.058
Don't know responses %	569 (77)		818 (62.5)		4756 (82.9)		74.8	(60.6,86.6)	234.8	2	<0.001	99.1	0.071
**INCLUDES DON'T KNOW AS NO**													
>10 Cell Counts	No		Yes		No								
% agreement	**		34.5		6.9		16.3	(3.2,36.7)	560.3	2	<0.001	99.6%	0.172
Sensitivity	**	**	31.2	28.6, 34	5.1	4.5, 5.7	7.6	(0.0,27.8)	704.1	2	<0.001	99.7%	0.235
Specificity	**	**	72.8	63.2, 81.1	96.4	91, 99	93.4	(72.5,100.0)	51.3	2	<0.001	96.1%	0.245
Positive Predictive Value %	**	**	93	90.1, 95.3	98.6	96.5, 99.6	95.8	(94.2,97.2)	-	-	<0.001	-	-
Negative Predictive Value %	**	**	8.4	6.6, 10.4	2	1.6, 2.4	6.7	(1.4,15.4)	167.4	2	<0.001	98.8%	0.061
AUC	**	**	0.5	0.5, 0.6	0.5	0.5, 0.5							
Inflation Factor	**		0.34		0.05								
**Cesarean Births**													
Observer Prevalence %	1646 (77.2)		930 (96)		1023 (97.9)		92.2	(75.5,99.8)	440.7	2	<0.001	99.5%	0.171

Survey-Reported Prevalence %	1 (0)		186 (19.7)		26 (2.6)		4.6	(0.0,20.5)	489.9	2	<0.001	99.5%	0.198
Don't know responses %	1630 (78.1)		664 (70.3)		863 (87.8)		79.5	(69.5,87.9)	100.9	2	<0.001	98.0%	0.040
**INCLUDES DON'T KNOW AS NO**													
>10 Cell Counts	No		No		No								
% agreement	22.5		22.5		4.1		15.0	(4.8,29.5)	229.9	2	<0.001	99.1%	0.096
Sensitivity	0.1	0, 0.3	20	17.4, 22.7	2.3	1.4, 3.5	4.5	(0.0,20.6)	439.5	2	<0.001	99.5%	0.203
Specificity	100	99.2, 100	86.1	70.5, 95.3	100	79.4, 100	98.2	(83.6,100.0)	19.1	2	<0.001	89.5%	0.175
Positive Predictive Value %	100	2.5, 100	97.3	93.8, 99.1	100	83.2, 100	100	(100.0,100.0)	59.2	2	<0.001	0.0%	0.0
Negative Predictive Value %	22.5	20.7, 24.3	4.1	2.8, 5.8	1.9	1.1, 3	7.6	(0.1,24.6)	390.5	2	<0.001	99.4%	0.179
AUC	0.5	0.5, 0.5	0.5	0.5, 0.6	0.5	0.5, 0.5							
Inflation Factor	0.00		0.21		0.02								
**Anything Applied to the Cord**													
**All modes of birth Combined**													
Observer Prevalence %	2588 (89.4)		2260 (97.9)		7123 (99.3)		96.5	(89.0,99.9)	503.6	2	<0.001	99.6%	0.071
Survey-Reported Prevalence %	87 (3.1)		1393 (61.8)		631 (9.4)		20.1	(0.7,55.8)	3075.6	2	<0.001	99.9%	0.448
Don't know responses %	2115 (74.8)		698 (31)		5328 (79)		62.5	(34.1,86.8)	1775.8	2	<0.001	99.9%	0.259

**INCLUDES DON'T KNOW AS NO**													
>10 Cell Counts	No		Yes		No								
% agreement	12.9		61.6		9.7		25.5	(3.7,57.8)	2376.9	2	<0.001	99.9%	0.349
Sensitivity	3.1	2.4, 3.8	62.1	60, 64.1	9.4	8.7, 10.2	20.1	(0.7,56.3)	2969.3	2	<0.001	99.9%	0.458
Specificity	96.9	94.3, 98.6	38.6	24.4, 54.5	91.7	73, 99	80.6	(35.3,100.0)	79.71	2	<0.001	97.5%	0.660
Positive Predictive Value %	89.7	81.3, 95.2	98.1	97.2, 98.7	99.7	98.8, 100	97.6	(93.6,99.7)	26.3	2	<0.001	92.4%	0.025
Negative Predictive Value %	10.4	9.3, 11.6	2	1.2, 3.2	0.4	0.2, 0.6	3.1	(0.0,12.7)	536.4	2	<0.001	99.6%	0.108
AUC	0.5	0.5, 0.5	0.5	0.4, 0.6	0.5	0.5, 0.6							
Inflation Factor	0.03		0.63		0.09								
**Vaginal Births**													
Observer Prevalence %	732 (96.3)		1293 (96.7)		6085 (99.3)		97.7	(94.7,99.5)	69.322	2	<0.001	97.1%	0.020
Survey-Reported Prevalence %	14 (1.9)		915 (69.9)		559 (9.7)		21.6	(0.0,65.8)	2178.1	2	<0.001	99.9%	0.667
Don't know responses %	555 (75.1)		315 (24.1)		4485 (78.1)		59.7	(23.9,90.4)	1409.5	2	<0.001	99.8%	0.432
**INCLUDES DON'T KNOW AS NO**													
>10 Cell Counts	No		Yes		No								
% agreement	5.2		69.6		10.1		24.1	(0.1,66.8)	1987.9	2	<0.001	99.8%	0.611
Sensitivity	1.8	1, 3.1	70.7	68.1, 73.2	9.8	9, 10.6	21.7	(0.0,66.3)	2142.9	2	<0.001	99.9%	0.678
Specificity	96.2	80.4, 99.9	38.1	23.6, 54.4	90.9	70.8, 98.9	78.4	(34.6,100.0)	35.7	2	<0.001	94.4%	0.576

Positive Predictive Value %	92.9	66.1, 99.8	97.2	95.9, 98.1	99.6	98.7, 100	99.0	(95.4,100.0)	17.9	2	<0.001	88.2%	0.021
Negative Predictive Value %	3.5	2.2, 5.1	4.2	2.4, 6.7	0.4	0.2, 0.6	2.2	(0.2,6.3)	64.271	2	<0.001	96.9	0.033
AUC	0.5	0.5, 0.5	0.5	0.5, 0.6	0.5	0.4, 0.6							
Inflation Factor	0.02		0.73		0.10								
**Cesarean Births**													
Observer Prevalence %	1855 (86.9)		967 (99.6)		1038 (99)		96.8	(85.8,100)	337.0	2	<0.001	99.4%	0.131
Survey-Reported Prevalence %	73 (3.5)		478 (50.6)		70 (7.1)		16.4	(0.3,48.9)	979.0	2	<0.001	99.8%	0.397
Don't know responses %	1560 (74.8)		383 (40.6)		819 (83.3)		67.5	(43.1,87.7)	470.4	2	<0.001	99.5%	0.190
**INCLUDES DON'T KNOW AS NO**													
>10 Cell Counts	No		No		No								
% agreement	15.6		50.6		7.4		22.2	(4.6,48.0)	557.5	2	<0.001	99.4%	0.234
Sensitivity	3.6	2.8, 4.5	50.6	47.4, 53.9	7.2	5.5, 9.1	16.5	(0.3,49.6)	935.5	2	<0.001	99.7%	0.410
Specificity	97	94.2, 98.7	50	1.3, 98.7	100	15.8, 100	98.5	(66.9,100.0)	3.7	2	<0.001	47.3%	0.182
Positive Predictive Value %	89	79.5, 95.1	99.8	98.8, 100	100	94.3, 100	98.2	(90.1,100.0)	21.7	2	<0.001	90.7%	0.086
Negative Predictive Value %	12.9	11.5, 14.5	0.2	0, 1.2	0.2	0, 0.9	2.5	(0.0,14.7)	307.1	2	<0.001	99.3%	0.169
AUC	0.5	0.5, 0.5	0.5	0, 1	0.5	0.5, 0.5							
Inflation Factor	0.04		0.51		0.07								

n=12,379 observed livebirths, n=11,827 livebirths with survey

*Red colour: cell count is <5 in 2x2 table; ** indicates 2x2 table could not be constructed; Green colour: cell count is ≥5 but <10 in 2x2 table*
