# Supplementary material for: Chlorhexidine for facility-based umbilical cord care: EN-BIRTH multi-country validation study
Source: BMC Pregnancy Childbirth. 2021 Mar 26;21(Suppl 1):239. doi: 10.1186/s12884-020-03338-4 (PMC7995704; doi:10.1186/s12884-020-03338-4)
Supplement: Supplementary file 7 — Additional file 7. Exit-survey reported health-worker communication of Chlorhexidine application, EN-BIRTH study (n = 11,639 live births). [file 12884_2020_3338_MOESM7_ESM.pdf]

SUPPLEMENT TITLE:

Every Newborn BIRTH multi-country validation study: informing measurement of coverage and quality of maternal and newborn care

PAPER TITLE:

Chlorhexidine for facility-based umbilical cord care: EN-BIRTH multi-country validation study

Additional File 7: Exit-survey reported health-worker communication of Chlorhexidine application, EN-BIRTH study (n=11,639)

	Azimpur Tertiary			Kushtia District			Pokhara Regional		
	Vaginal births	Caesarean	Total	Vaginal births	Caesarean	Total	Vaginal births	Caesarean	Total
	n (%)	n (%)	n (%)	n (%)	n (%)	n (%)	n (%)	n (%)	n (%)
**The person that applied chlorhexidine**									
Health worker	4 (0.5)	7 (0.3)	11 (0.4)	596 (45.5)	231 (24.5)	827 (36.7)	533 (9.3)	64 (6.5)	599 (8.9)
Mother	0 (0)	0 (0)	0 (0)	0 (0)	1 (0.1)	1 (0)	0 (0)	0 (0)	0 (0)
Not applied	169 (22.9)	454 (21.8)	624 (22.1)	75 (5.7)	82 (8.7)	157 (7)	679 (11.8)	88 (9)	767 (11.4)
Don't know	565 (76.5)	1625 (77.9)	2190 (77.5)	634 (48.4)	629 (66.6)	1263 (56.1)	4511 (78.6)	825 (83.9)	5360 (79.4)
**Respondents were told the reason for CHX application**									
Yes	1 (0.1)	2 (0.1)	3 (0.1)	80 (6.1)	47 (5)	127 (5.6)	73 (1.3)	7 (0.7)	80 (1.2)
No	1 (0.1)	2 (0.1)	3 (0.1)	266 (20.3)	110 (11.7)	376 (16.7)	431 (7.5)	55 (5.6)	488 (7.2)
Not applied	169 (22.9)	454 (21.8)	624 (22.1)	75 (5.7)	82 (8.7)	157 (7)	679 (11.8)	88 (9)	767 (11.4)
Don't know	567 (76.9)	1628 (78)	2195 (77.7)	884 (67.5)	704 (74.6)	1588 (70.5)	4540 (79.1)	827 (84.1)	5391 (79.9)

**Total includes vaginal and caesarean*

n= Number; %=Percentage; CHX: Chlorhexidine
