# Supplementary material for: Chlorhexidine for facility-based umbilical cord care: EN-BIRTH multi-country validation study
Source: BMC Pregnancy Childbirth. 2021 Mar 26;21(Suppl 1):239. doi: 10.1186/s12884-020-03338-4 (PMC7995704; doi:10.1186/s12884-020-03338-4)
Supplement: Supplementary file 8 — Additional file 8. Validation register-recorded umbilical cord care practices, EN-BIRTH study (n = 10,772). [file 12884_2020_3338_MOESM8_ESM.pdf]

SUPPLEMENT TITLE:

Every Newborn BIRTH multi-country validation study: informing measurement of coverage and quality of maternal and newborn care

PAPER TITLE:

Chlorhexidine for facility-based umbilical cord care: EN-BIRTH multi-country validation study

Additional File 8: Validation register-recorded umbilical cord care practices, EN-BIRTH study (n=10,772)

	**Azimpur (BD)**		**Kushtia (BD)**		**Pokhara (NP)**		**Overall**						
	Tertiary		District		Regional		Pooled Random Effects						
	N(%)	(CI)	N(%)	(CI)	N(%)	(CI)	%	(CI)	**Q**	**df**	**p-val.**	**i^2**	**τ^2**
**Register-Recorded Denominator**	2222 livebirths		1839 livebirths		6711 livebirths		10772 livebirths						
**All modes of birth Combined**													
Observer Prevalence %	2582 (89.3)		2257 (97.9)		7112 (99.4)		99.6	(88.8,99.9)	528.1	2	<0.001	99.6%	0.074
Register-Recorded Prevalence %	2185 (98.3)		1796 (97.7)		5282 (78.7)		93.7	(76.4,100.0)	1162.1	2	<0.001	99.8%	0.199
Not recorded	13 (0.6)		41 (2.2)		1394 (20.8)		5.4	(0.0,23.5)	1350.2	2	<0.001	99.8%	0.232
Not readable	0 (0)		0 (0)		4 (0.1)		0.0	(0.0,0.1)	2.214	2	<0.001	9.6%	<0.001
**INCLUDES NOT RECORDED AND NOT READABLE AS NO**													
>10 Cell Counts	No		No		Yes								
% agreement	88.6		96.4		78.7		89.0	(76.4,97.1)	514.3	2	<0.001	99.6%	0.088
Sensitivity	98.3	97.7, 98.9	97.8	97.1, 98.5	79	78, 80	93.8	(76.7,100.0)	1097.8	2	<0.001	99.8%	0.198
Specificity	1.8	0.5, 4.5	6.9	0.8, 22.8	25	12.7, 41.2	8.8	(0.0,0.280)	21.67	2	<0.001	90.7%	0.171

Positive Predictive Value %	89.9	88.5, 91.1	98.5	97.8, 99	99.4	99.2, 99.6	97.0	(89.8,100.0)	383.0	2	<0.001	99.4%	0.071
Negative Predictive Value %	10.8	3, 25.4	4.9	0.6, 16.5	0.7	0.3, 1.3	3.8	(0.0,13.7)	13.8	2	<0.001	85.6%	0.094
AUC	0.5	0.5, 0.5	0.5	0.5, 0.6	0.5	0.5, 0.6							
Inflation Factor	1.09		0.99		0.79								
**EXCLUDES NOT RECORDED AND NOT READABLE**													
>10 Cell Counts	No		No		No								
% agreement	88.6		96.4		98.9		95.5	(87.7,99.5)	365.3	2	<0.001	99.4%	0.065
Sensitivity	98.3	97.7, 98.9	97.8	97.1, 98.5	99.4	99.2, 99.6	98.6	(97.3,99.5)	35.8	2	<0.001	94.4%	0.006
Specificity	1.8	0.5, 4.5	6.9	0.8, 22.8	0	0, 11.6	1.5	(0.0,5.0)	2.8	2	<0.001	30.0%	0.009
Positive Predictive Value %	89.9	88.5, 91.1	98.5	97.8, 99	99.4	99.2, 99.6	97.0	(89.8,100.0)	383.0	2	<0.001	99.4%	0.071
Negative Predictive Value %	10.8	3, 25.4	4.9	0.6, 16.5	0	0, 11.6	10.8	(0.0,12.4)	4.5	2	<0.001	56.3%	0.039
AUC	0.5	0.5, 0.5	0.5	0.5, 0.6	0.5	0.5, 0.5							
Inflation Factor	1.09		0.99		1.00								
**Vaginal Births**													
Observer Prevalence %	731 (96.3)		1290 (96.5)		6075 (99.4)		97.7	(94.4,99.6)	78.5	2	<0.001	97.4%	0.023
Register-Recorded Prevalence %	547 (97.9)		1073 (97.6)		4963 (84.3)		94.5	(82.2,99.9)	334.0	2	<0.001	99.4%	0.121
Not recorded	7 (1.3)		25 (2.3)		894 (15.2)		4.9	(0.0,17.1)	351.7	2	<0.001	99.4%	0.127
Not readable	0 (0)		0 (0)		3 (0.1)		0.0	(0.0,0.1)	0.323	2	0.851	0.0%	<0.001
**INCLUDES NOT RECORDED AND NOT READABLE AS NO**													

>10 Cell Counts	No		No		No								
% agreement	94.4		95.4		83.9		91.9	(82.1,98.0)	184.0	2	<0.001	98.9%	0.066
Sensitivity	98	96.4, 99	97.8	96.8, 98.6	84.3	83.4, 85.3	94.6	(82.2,99.9)	337.8	2	<0.001	99.4%	0.126
Specificity	4.8	0.1, 23.8	6.9	0.8, 22.8	12.5	3.5, 29	8.3	(2.8,15.7)	83.0	2	0.660	0.0%	<0.001
Positive Predictive Value %	96.3	94.4, 97.7	97.5	96.4, 98.3	99.4	99.2, 99.6	98.0	(95.4,99.6)	46.9	2	<0.001	95.7%	0.017
Negative Predictive Value %	8.3	0.2, 38.5	8	1, 26	0.4	0.1, 1.1	2.8	(0.0,15.3)	9.3	2	0.009	78.6%	0.280
AUC	0.5	0.5, 0.6	0.5	0.5, 0.6	0.5	0.4, 0.5							
Inflation Factor	1.02		1.00		0.85								
**EXCLUDES NOT RECORDED AND NOT READABLE**													
>10 Cell Counts	No		No		No								
% agreement	94.4		95.4		98.9		96.6	(92.5,99.1)	68.1	2	<0.001	97.0%	0.025
Sensitivity	98	96.4, 99	97.8	96.8, 98.6	99.4	99.2, 99.6	98.6	(96.9,99.6)	24.3	2	<0.001	91.7%	0.009
Specificity	4.8	0.1, 23.8	6.9	0.8, 22.8	0	0, 12.3	2.9	(0.0,9.5)	2.5	2	0.660	21.6%	0.011
Positive Predictive Value %	96.3	94.4, 97.7	97.5	96.4, 98.3	99.4	99.2, 99.6	98.0	(95.4,99.6)	46.9	2	<0.001	95.7%	0.017
Negative Predictive Value %	8.3	0.2, 38.5	8	1, 26	0	0, 11.9	3.3	(0.0,13.2)	3.5	2	<0.001	44.1%	0.037
AUC	0.5	0.5, 0.6	0.5	0.5, 0.6	0.5	0.5, 0.5							
Inflation Factor	1.02		1.00		1.00								
**Cesarean Births**													
Observer Prevalence %	1850 (86.8)		967 (99.8)		1037 (99.2)		97.1	(85.6,100.0)	379.5	2	<0.001	99.4%	0.147
Register-Recorded Prevalence %	1638 (98.5)		723 (97.7)		318 (39)		85.5	(41.5,100.0)	1421.4	2	<0.001	99.8%	0.715

Not recorded	6 (0.4)		16 (2.2)		495 (60.7)		12.9	(0.0,58.9)	1588.9	2	<0.001	99.8%	0.800
Not readable	0 (0)		0 (0)		1 (0.1)		0.0	(0.0,0.1)	2.0	2	0.367	0.1%	<0.001
**INCLUDES NOT RECORDED AND NOT READABLE AS NO**													
>10 Cell Counts	No		No		No								
% agreement	86.6		**		40.2		79.5	(43.2,99.3)	902.6	2	<0.001	99.7%	0.459
Sensitivity	98.5	97.7, 99.1	**	**	39.8	36.4, 43.4	85.8	(42.4,100.0)	1322.0	2	<0.001	99.8%	0.702
Specificity	1.5	0.3, 4.2	**	**	75	34.9, 96.8	0.6	(0.0,2.9)	-	-	0.2	-	-
Positive Predictive Value %	87.7	86, 89.3	**	**	99.4	97.7, 99.9	97.7	(85.1,100.0)	262.3	2	<0.001	99.2%	0.181
Negative Predictive Value %	12	2.5, 31.2	**	**	1.3	0.5, 2.7	2.2	(0.0,10.9)	6.4	2	0.039	69.0%	0.057
AUC	0.5	0.5, 0.5	**	**	0.6	0.4, 0.7							
Inflation Factor	1.12		**		0.40								
**EXCLUDES NOT RECORDED AND NOT READABLE**													
>10 Cell Counts	No		No		No								
% agreement	86.6		**		99.1		95.7	(85.5,100.0)	149.4	2	<0.001	98.6%	0.101
Sensitivity	98.5	97.7, 99.1	**	**	99.7	98.2, 100	98.7	(97.6,99.4)	5.9	2	0.051	66.3%	0.003
Specificity	1.5	0.3, 4.2	**	**	0	0, 84.2	0.0	(0.0,0.0)	-	-	1.0	-	-
Positive Predictive Value %	87.7	86, 89.3	**	**	99.4	97.7, 99.9	97.7	(85.1,100.0)	262.3	2	<0.001	99.2%	0.181
Negative Predictive Value %	12	2.5, 31.2	**	**	0	0, 97.5	0.0	(0.0,11.0)	2.63	2	0.268	23.9	0.028

AUC	0.5	0.5, 0.5	**	**	0.5	0.5, 0.5	
Inflation Factor	1.12		**		1.00		

*Red colour: cell count is <5 in 2x2 table; ** indicates 2x2 table could not be constructed; Green colour: cell count is ≥5 but <10 in 2x2 table*
